# Machine Learning to Enhance Electronic Detection of Diagnostic Errors

**DOI:** 10.1001/jamanetworkopen.2024.31982

**Published:** 2024-09-09

**Authors:** Andrew J. Zimolzak, Li Wei, Usman Mir, Ashish Gupta, Viralkumar Vaghani, Devika Subramanian, Hardeep Singh

**Affiliations:** 1 Center for Innovations in Quality, Effectiveness and Safety, Michael E. DeBakey Veterans Affairs Medical Center and Baylor College of Medicine, Houston, Texas; 2 Department of Medicine, Baylor College of Medicine, Houston, Texas; 3 Department of Computer Science, Rice University, Houston, Texas

## Abstract

This cohort study examines whether machine learning (ML) can enhance the ability of electronic triggers to identify possible missed opportunities in diagnosis.

## Introduction

Diagnostic errors contribute substantially to patient harm, but strategies to monitor them are underdeveloped.^1^ Electronic trigger algorithms (e-triggers) can identify patients with potential diagnostic errors^2^ using electronic health record (EHR) data. However, their predictive values are low, and this process requires time-consuming manual medical record review to confirm missed opportunities in diagnosis (MODs).^3^ Because e-triggers are designed using a priori assumptions rather than empirical data patterns, they may not detect MOD signals comprehensively. We tested whether machine learning (ML) can enhance e-trigger performance and emulate human medical record reviewers at a larger scale.^4^

## Methods

Based on expert input and existing frameworks,^2,5^ we designed rules-based e-triggers to find possible MODs in emergency departments (ED). Using Veterans Affairs national EHR data covering more than 20 million unique individuals, we identified 2 high-risk cohorts: (1) patients with stroke risk factors discharged from ED after presenting with dizziness or vertigo who were subsequently hospitalized for stroke or TIA within 30 days; and (2) patients discharged from ED with abdominal pain and abnormal temperature who were subsequently hospitalized within 10 days. All ED visits occurred between 2016 and 2020. Trained clinicians used standardized data collection instruments (eFigure 1 in Supplement 1) to review a random sample of medical records flagged by each e-trigger and labeled each as MOD or no MOD. Baylor College of Medicine review board approved the study and granted waiver of informed consent because it would not be feasible to obtain consent for medical record reviews from the large number of patients that we studied. Analyses were conducted from April 2020 to May 2024 using Python version 3.7.4 (Python Software Foundation), with the packages scipy, numpy, and scikit-learn.

Medical records with clear evidence of MOD or no MOD were divided into training and test sets (eFigure 2 in Supplement 1). ML methods were regularized logistic regression and random forests (with limited maximum tree depth to mitigate overfitting). The dizziness and abdominal pain algorithms had access to 148 and 153 variables potentially associated with the outcomes, respectively, extracted from structured EHR data. These included demographics, laboratory values, vital signs, orders, visit times, and risk factors (eTable in Supplement 1). Because methods emulated retrospective medical record review evaluation, rather than prehospital^6^ or ED point-of-care evaluation, variables were drawn from index ED data and subsequent hospital data. Variables were preselected based on bivariate association with MOD by *t* test or χ^2^ test as appropriate, with a statistical significance threshold of 2-sided *P* = .10. Positive predictive values (PPV) are reported as pooled values (training and test set combined) due to the limited number of criterion standard records labeled by clinicians. CIs are 95% Wald intervals.

## Results

For the dizziness e-trigger, reviewers identified MODs in 39 of 82 flagged records (PPV, 48% [95% CI, 37%-58%]). The best-performing ML algorithm (random forest) correctly identified 36 of 39 true MODs and 40 of 43 negative MODs (PPV, 92% [95% CI, 84%-100%]). For the abdominal pain e-trigger, reviewers identified 31 MODs in 104 flagged records (PPV, 30% [95% CI, 21%-39%]). Examples of diagnostic errors included missed diagnoses of cholangitis, cholecystitis, and infectious colitis. ML correctly identified 26 of 31 true MODs and 71 of 73 negative MODs (PPV, 93% [95% CI, 83%-100%]). Details are shown in the Figure and Table.

**Figure.**
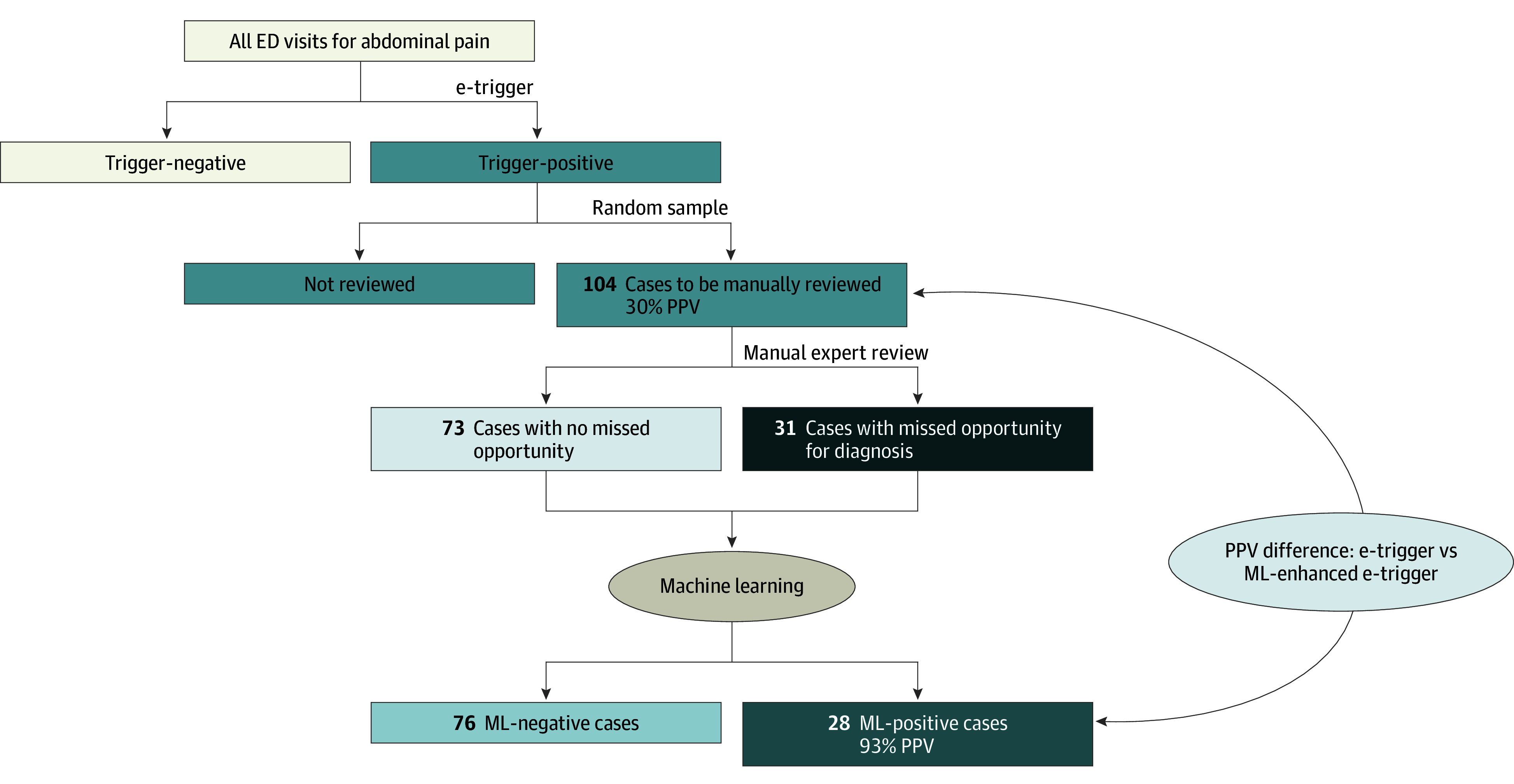
Example Study Flow Diagram A rules-based e-trigger was designed to identify patients presenting with abdominal pain, abnormal temperature, and a near future hospitalization. Experts reviewed a random sample from all trigger-positive cases to identify missed opportunities in diagnosis. All labeled cases had structured health record data analyzed by machine learning, aiming to separate true and false missed opportunities automatically. ED indicates emergency department; e-trigger, electronic trigger algorithm; ML, machine learning; PPV, positive predictive value.

**Table.** Predictive Value of Rules-Based and Machine Learning–Enhanced Electronic Triggers

Electronic trigger type	MOD rate by criterion standard	
	No. of medical records with MOD/total No. of medical records	Positive predictive value, % (95% CI)
**Dizziness**		
Rules-based positive for MOD	39/82	48 (37-58)
ML positive for MOD	36/39	92 (84-100)
ML negative for MOD	3/43	NA
**Abdominal pain**		
Rules-based positive for MOD	31/104	30 (21-39)
ML positive for MOD	26/28	93 (83-100)
ML negative for MOD	5/76	NA

Abbreviations: ML, machine learning; MOD, missed opportunity in diagnosis; NA, not applicable.

## Discussion

Machine learning enhanced the accuracy of electronic triggers to identify MODs. This ML enhancement could advance an organization’s ability to monitor diagnostic errors for research, learning, and quality improvement. Moreover, it substantially reduces the burden of clinician-dependent manual medical record review. Limitations of this study include the time needed to prepare the variables used by ML, although once this is done, the algorithm can run at a large scale. The small number of expert-labeled records may limit the ability of ML to use all structured data and to estimate test set performance.

Next steps include incorporating clinical note text as a source of missed opportunity prediction to leverage the rich clinical data needed to determine MODs, increasing the number of expert-labeled records on which the approach is tested, and validation in an external, independent population. Machine learning shows promise as a tool to efficiently identify diagnostic errors for research and quality improvement.

## Notes

Supplement 1. **eFigure 1. **Abdominal and Dizziness Data Collection Instruments **eFigure 2. **Flow of Records Including Random Sampling **eTable.** Machine Learning Values

Supplement 2.
Data Sharing Statement
