# Supplementary material for: Machine Learning to Enhance Electronic Detection of Diagnostic Errors
Source: JAMA Netw Open. 2024 Sep 9;7(9):e2431982. doi: 10.1001/jamanetworkopen.2024.31982 (PMC11385053; doi:10.1001/jamanetworkopen.2024.31982)
Supplement: Supplement 1. — eFigure 1. Abdominal and Dizziness Data Collection Instruments eFigure 2. Flow of Records Including Random Sampling eTable. Machine Learning Values [file jamanetwopen-e2431982-s001.pdf]

Supplemental Online Content

Zimolzak AJ, Wei L, Mir U, et al. Machine learning to enhance electronic detection of diagnostic errors. *JAMA Netw Open.* 2024;7(9):e2431982. doi:10.1001/jamanetworkopen.2024.31982

eFigure 1. Abdominal and Dizziness Data Collection Instruments

eFigure 2. Flow of Records Including Random Sampling

eTable 1. Machine Learning Values

This supplemental material has been provided by the authors to give readers additional information about their work.

© 2024 Zimolzak AJ et al. *JAMA Network Open.*

eFigure 1: Abdominal and dizziness data collection instruments (10 pp.)

HighRisk Abdominal Pain Data Collection Instrument

Study ID: ______ Reviewer Initials: ________ Review Date: _______

Trigger Record Details

1) **MRN: __________**
2) **Facility Information: __________**
3) **Index ER visit date: __________**
4) **Inpatient admission date: __________**
5) **Discharge date: ______________**
6) **Patient demographic information**
   a. Gender: *Male |Female |Other|….*
   b. Race: *White |African American |Asian|…….*
   c. Ethnicity: *Hispanic | non-Hispanic |….*
   d. Date of birth: __________
   e. Age at Index ER visit date: __________
   f. Zip code: __________
   g. Marital status: __________
   h. Homeless status: __________
   i. Socio-economic status: __________
7) **Elixhauser comorbidity Index:** ______

1. Trigger Inclusion Criteria

1) **Was patient admitted during index ER visit?** Yes |No
2) **Did patient have abdominal pain at Index ER visit?** Yes |No
3) **Did patient have fever (T>99.6 F or >37.5 C) or low body temp(T<96.8 F or <36 C) at Index ER visit?** Yes |No
4) **Did patient have inpatient admission within 10 days of Index ER visit?** Yes |No

2. Trigger Exclusion Criteria

1) **Was the patient in hospice or palliative care within one year prior to Index ER visit?** Yes |No
2) **Did the patient have admission and discharge on the same day?** Yes |No
3) **Did the patient have following terminal illness within one year prior to Index ER visit?**
   Lung Cancer |Brain Cancer |Liver Cancer |Pancreatic Cancer |Myeloma|…………

© 2024 Zimolzak AJ et al. *JAMA Network Open*.

4) **Was the patient admitted due to external causes (accident/Trauma/External Injuries/Burns/Poisoning etc.)?** Yes |No
5) **Was the patient admitted to Psych floor?** Yes |No
6) **Patient's age <18 or >85** Yes |No

3. Inpatient admission-Discharge details

1) **Inpatient admission date:** __________
   Type of inpatient admission: Planned |Unplanned| No admission
   Primary admission diagnosis: ______________
   Additional admission diagnosis: ______________
2) **Date of Discharge:** _______________
   Primary discharge diagnosis: ______________
   Additional discharge diagnosis: _______________

4. Index ER visit

1) **Index ER visit date:** ____________
2) **Number of ER visits in prior 3 months:** __________
3) **Number of hospitalizations in prior 1 year:** ___________
4) **Type of provider at Index ER visit:** MD |DO |NP |PA |Resident |Intern |Unable to determine
5) **Index ER visit chief complaint:** _____________
6) **Sign and symptoms at Index visit:**
   a. Abdominal pain details
      i. Onset: Sudden | Gradual |Not recorded ….
      ii. Duration: <24 hours|24-72 hours|72 hours-1week|….
      iii. Location: RUQ|RLQ|……
      iv. Radiation of pain: No| Not recorded |Yes
         a) If yes, where? _____________
      v. Quality of pain: _____________
      vi. Severity of pain at Index visit (1-10): _________
      vii. If any abdominal pain provoking factors: __________
      viii. If any abdominal pain palliating factors: ___________
   b. Associated symptoms: ____________________
7) **Past Medical History at Index visit:**
   a. History of abdominal surgery: Yes| No
      i. If yes, please specify: ______________
   b. History of mental health disorder on treatment (in history list or prior visits to psych): Yes | No
      i. If yes, please specify mental disorders: ____________
   c. History of substance abuse disorder (in history list, social history or prior visits): Yes |No
      i. If yes, please specify: ____________ (alcohol, tobacco, opiates, other substances)
         a) Please specify "other substances": _____________
8) **Physical examination at Index visit:**
   a. Vitals:

i. BMI
ii. Blood pressure
iii. Temperature
iv. Heart rate
v. Respiratory rate

b. Abdominal examination:
   i. General Inspection: Not recorded | Surgical scars |Skin discoloration |Others
   ii. Guarding: Yes | No | Not recorded
   iii. Rigidity: Yes | No | Not recorded
   iv. Rebound Tenderness: Yes | No | Not recorded
   v. Localized Tenderness: Yes | No | Not recorded
   vi. Positive signs: None| Murphy Sign |Psoas Sign| Obturator sign |……
   vii. Rectal examination: Yes | No | Not recorded
   viii. Any other relevant abnormal findings: _____________

9) Diagnostic Evaluation at Index visit

   i. Ordered lab tests: CBC |BMP |LFTs| Cardiac Biomarkers |Coagulation studies | ABG |UA |UC |FOBT| Other
      a) What lab abnormality? ___________________
   ii. Ordered l Imaging tests: X-ray |CT scan |MRI |USG|….
      a) What abnormality? ____________
   iii. Ordered consults: GI |Oncology|….
   iv. Did ED provider review abnormal lab or imaging findings prior to discharge? Yes |No |Unsure

10) Assessment at Index visit

   a. Primary diagnosis in the assessment/plan: ________
   b. Additional diagnoses in the assessment/plan: __________

11) Did patient leave against medical advice? Yes| No

12) Number of ER or PCP visits between Index visit and hospitalization for abdominal pain:

____

5. Safer Dx Instrument Opportunity

Rate the following items for the episode of care under review:

1—2—3—4—5—6—7

1 = Strongly Disagree 7 = Strongly Agree

© 2024 Zimolzak AJ et al. JAMA Network Open.

	Item	Score
1.	The documented history was suggestive of an alternate diagnosis, which was not considered in the diagnostic process.	
2.	The documented physical exam was suggestive of an alternate diagnosis, which was not considered in the diagnostic process. *	
3.	Data gathering through history, physical exam, and review of prior documentation (including prior laboratory, radiology, pathology or other results) was incomplete, given the patient's medical history and clinical presentation.	
4.	Alarm symptoms or "Red Flags" (i.e., features in the clinical presentation that are considered to predict serious disease) were not acted upon.	
5.	The diagnostic process was affected by incomplete or incorrect clinical information given to the care team by the patient or their primary caregiver.	
6.	The clinical information (i.e., history, physical exam or diagnostic data) should have prompted additional diagnostic evaluation through tests or consults.	
7.	The diagnostic reasoning was not appropriate, given the patient's medical history and clinical presentation.	
8.	Diagnostic data (laboratory, radiology, pathology or other results) available or documented were misinterpreted in relation to the subsequent final diagnosis.	
9.	There was missed follow-up of available or documented diagnostic data (laboratory, radiology, pathology or other results) in relation to the subsequent final diagnosis.	
10.	The differential diagnosis was not documented OR the documented differential diagnosis did not include the subsequent final diagnosis.	
11.	The final diagnosis was not an evolution of the care team's initial presumed diagnosis (or working diagnosis).	
12.	The clinical presentation at the initial or subsequent presentation was mostly typical of the final diagnosis.	
13.	In conclusion, based on all the above questions, the episode of care under review has a missed opportunity to make a correct and timely diagnosis.	

* *Physical exam includes vital signs*

Additional information, please check "Yes" if applicable:

1. Care episode involves a management error. ☐ Yes
2. Care escalation (e.g., hospitalization at subsequent visit) was related to worsening of an original correctly diagnosed condition that the patient initially presented with (rather than from something being missed initially) ☐ Yes

Please fill the process breakdown form if any missed opportunity of diagnosis (Score >5 on Safer Dx Item13)

6. Process Breakdown form

Outcome

What was the potential severity of injury associated with delay or missed diagnosis? (Select one)

Death |Severe harm | Moderate harm | Mild harm |No harm | Unknown

Dimensions

© 2024 Zimolzak AJ et al. *JAMA Network Open.*

If there was a missed opportunity of diagnosis, to which of the following dimensions can it be attributed (select all that apply)?

Patient:

1. Delay in seeking care
2. Lack of adherence to appointments
3. Other

If other, please specify here: ________________

Patient-Provider Encounter:

1. Problems with history.
2. Problems with physical exam.
3. Problems ordering diagnostic tests.
4. Failure to review previous documentation
5. Problems with data integration and interpretation
6. Other

If other, please specify here: ________________

Diagnostic Tests:

1. Ordered test not performed at all.
2. Ordered tests not performed correctly.
3. Performed tests not interpreted correctly.
4. Misidentification.
5. Other

If other, please specify here: ________________

Follow-up and Tracking:

1. Problems w/ timely FU of abnormal test results.
2. Problems w/ scheduling appropriate, timely FU visits.
3. Problems w/ diagnostic specialties returning test results.
4. Problems w/ reviewing test results.
5. Problems w/ documenting response to test results.
6. Problems w/ monitoring patients thru FU.
7. Other

If other, please specify here: ________________

Referrals:

1. Problem initiating referral.
2. Lack of appropriate actions on requested consultation.
3. Communication breakdown from consultant to referring provider.
4. Other

If other, please specify here: ________________

7. Case summary

© 2024 Zimolzak AJ et al. *JAMA Network Open.*

Case summary: Brief description of missed diagnostic opportunity or management error and any relevant thoughts and observations that helped with your decision (for or against).

--END---

HighRisk Dizziness Data Collection Instrument

Study ID: ______ Reviewer Initials: ________ Review Date: _______

1. Trigger Record Details

1) **MRN:** __________
2) **Facility Information:** __________
3) **Index ER visit date:** __________
4) **Inpatient admission date:** __________
5) **Discharge date:** ______________
6) **Patient demographic information**
   a. Gender: *Male |Female |Other|….*
   b. Race: *White |African American |Asian|…….*
   c. Ethnicity: *Hispanic | non-Hispanic |….*
   d. Date of birth: __________
   e. Age at Index ER visit date: __________
   f. Zip code: __________
   g. Marital status: __________
   h. Homeless status: __________
   i. Socio-economic status: __________
7) **Elixhauser comorbidity Index:** ______

2. Trigger Inclusion Criteria

1) **Was patient admitted during index ER visit?** Yes |No
2) **Did patient have dizziness related diagnosis at the Index ER visit?** Yes |No
3) **Did patient have two or more stroke risk factors at Index ER visit?** Yes |No
4) **Did patient have inpatient admission for Stroke or TIA within 30 days of Index ER visit?** Yes |No

If any answer is No, please directly go to "Type of Error" section.

3. Trigger Exclusion Criteria

© 2024 Zimolzak AJ et al. *JAMA Network Open.*

1) **Was the patient in hospice or palliative care within one year prior to Index ER visit?** Yes |No
2) **Did the patient have admission and discharge within 24 hours?** Yes |No
3) **Did the patient have following terminal illness within one year prior to Index ER visit?**
   Lung Cancer |Brain Cancer |Liver Cancer |Pancreatic Cancer |Myeloma|…………
4) **Was the patient admitted due to external causes (accident/Trauma/External Injuries/Burns/Poisoning etc.)?** Yes |No
5) **Was the patient admitted to Psych floor?** Yes |No

4. Inpatient admission

3) **Inpatient admission date: ___________**

   Primary admission diagnosis: ______________
   a. If any additional diagnoses, please specify: ______________
4) **Discharge date: _______________**
   Primary discharge diagnosis: ______________
   a. If any additional diagnoses, please specify: _______________
   Stroke description
   b. Type of stroke:
      TIA |Ischemic stroke |Hemorrhagic stroke |Unspecified/Inconclusive
   c. Location of stroke:

5. Index ER visit

1) **Number of ER visits in prior 3 months: __________**
2) Index ER visit date: _______________
3) **Type of provider at Index ER visit:** MD |DO |NP |PA |Resident |Intern |Unable to determine
4) **Describe chief complaints: _____________**

Stroke Risk factors:

a) History of Stroke or TIA: Yes | No
b) Current smoker: Yes | No
c) Hyperlipidemia: Yes | No
d) Diabetes Mellitus: Yes | No
e) Hypertension or Blood pressure >=140/90 at ER: Yes | No
f) Hx of Carotid stenosis: Yes | No
g) Hx of Atrial Fibrillation: Yes | No
h) Hx of Aneurysm: Yes | No
i) Hx of CAD: Yes| No

Stroke red flags

a) Diplopia: Yes | No

© 2024 Zimolzak AJ et al. *JAMA Network Open.*

b) Visual field defect (e.g., monocular vision loss, bilateral hemianopia etc.): Yes | No
c) Loss of consciousness/pre-syncope: Yes | No
d) Seizure: Yes | No
e) Speech Abnormalities: Yes | No
f) Unilateral limb weakness: Yes | No
   a. Unilateral arm weakness: Yes | No
   b. Unilateral leg weakness: Yes | No
g) UMN facial weakness: Yes | No
h) Duration of neurological symptoms
   i. ☐ <10 min ☐ 10-59 min ☐ >=60 min ☒ No symptoms
i) Hypoglycemia at ER: Yes | No
j) Sudden severe Headache Yes | No
k) Sudden confusion Yes | No
l) Sudden loss of balance/coordination Yes | No

Dizziness Evaluation

a. Is patient's age >60 years? Yes|No
b. Hx of recurrent isolated dizziness: Yes |No
c. Is dizziness symptom getting worse by triggers (Action, movements, or situations)? Yes |No
d. Associated symptoms: ___________________
e. Was patient on any medication commonly associated with dizziness? Yes |No

5) Physical examination at Index visit:

a. Vitals: (Reviewer doesn't have to fill vital information.)
   i. BMI
   ii. Blood pressure
   iii. Temperature
   iv. Heart rate
   v. Respiratory rate
b. Neurological examination:
   i. Mental status examination: Yes | No | Not recorded
   ii. Cranial Nerves examination Yes | No | Not recorded
   iii. Nystagmus testing: Yes | No | Not recorded
      a) If yes, please specify result: ____________
   iv. Sensory examination: Yes | No | Not recorded
   v. Motor examination: Yes | No | Not recorded
   vi. Reflex testing: Yes | No | Not recorded
   vii. Coordination testing: Yes | No | Not recorded
   viii. Gait testing: Yes | No | Not recorded
   ix. Dix-Hallpike maneuver (Specific for Dizziness): Positive |Negative | Not performed/Not noted

© 2024 Zimolzak AJ et al. *JAMA Network Open.*

x. HINTs Examination (Specific for Dizziness): Positive |Negative | Not performed/Not noted

TIA/Stroke predictor score (Dawson TIA score, ROSIER, and ABCD score will be automatically calculated the score based on TIA/Stroke evaluation)

Dawson TIA score: ____
ROSIER scale: ________
ABCD2 Score: __________
NIH stroke scale: Performed |Not performed

6) Diagnostic Evaluation at Index visit

i. Neurology consult: Yes| No
ii. MRI: Yes | No
  a. If yes, Result of MRI: ____
iii. CT: Yes |No
  a. If yes, Result of CT scan: _____
iv. Did ED provider review abnormal lab or imaging findings prior to discharge? Yes |No |Unsure

7) Assessment at Index visit

a. Primary diagnosis in the assessment/plan: ________
b. Additional diagnoses in the assessment/plan: __________

8) Did patient leave against medical advice? Yes |No

9) Number of ER visits between Index visit and hospitalization for dizziness: ___________

6. Type of Error

No missed opportunity | Potentially missed opportunity | Missed opportunity | Inconclusive | Coding error

7. Process Breakdown form (Only for MOD)

Harm Scale for MOD/PMOD:

Death |Severe harm | Moderate harm | Mild harm |No harm | Unknown

Dimensions

If there was a missed opportunity of diagnosis, to which of the following dimensions can it be attributed (select all that apply)?

Patient:

4. Delay in seeking care
5. Lack of adherence to appointments
6. Other

If other, please specify here: ________________

Patient-Provider Encounter:

7. Problems with history.

© 2024 Zimolzak AJ et al. *JAMA Network Open.*

8. Problems with physical exam.
9. Problems ordering diagnostic tests.
10. Failure to review previous documentation
11. Problems with data integration and interpretation
12. Other

If other, please specify here: ________________

Diagnostic Tests:

6. Ordered test not performed at all.
7. Ordered tests not performed correctly.
8. Performed tests not interpreted correctly.
9. Misidentification.
10. Other

If other, please specify here: ________________

Follow-up and Tracking:

8. Problems w/ timely FU of abnormal test results.
9. Problems w/ scheduling appropriate, timely FU visits.
10. Problems w/ diagnostic specialties returning test results.
11. Problems w/ reviewing test results.
12. Problems w/ documenting response to test results.
13. Problems w/ monitoring patients thru FU.
14. Other

If other, please specify here: ________________

Referrals:

5. Problem initiating referral.
6. Lack of appropriate actions on requested consultation.
7. Communication breakdown from consultant to referring provider.
8. Other

If other, please specify here: ________________

9. Case summary

Case summary:

---END---

© 2024 Zimolzak AJ et al. *JAMA Network Open.*

eFigure 2: Flow of records including random sampling

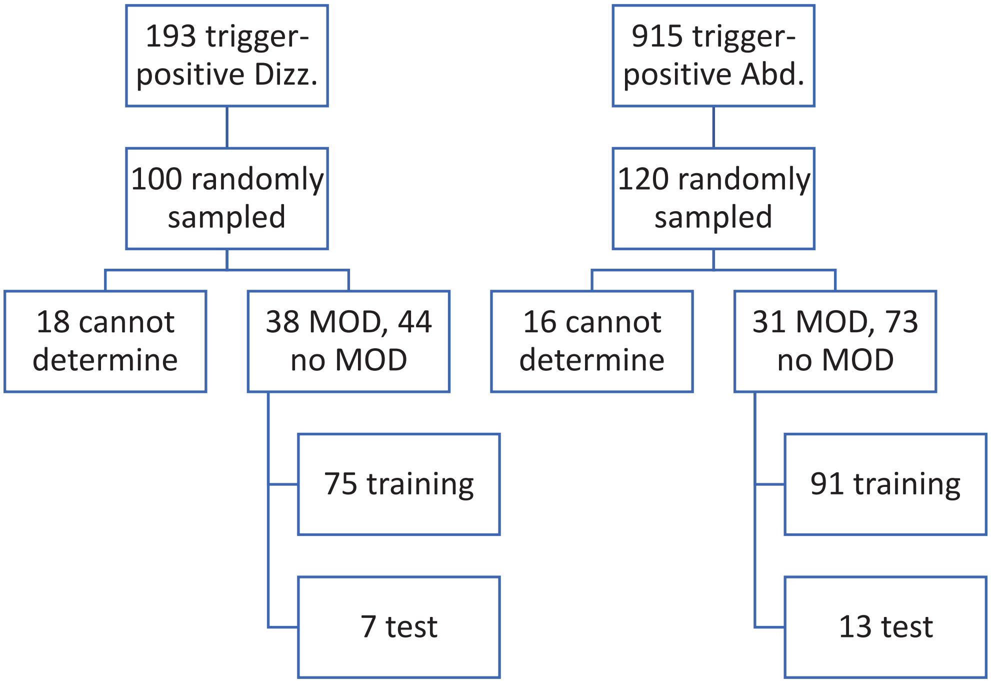

© 2024 Zimolzak AJ et al. *JAMA Network Open.*

eTable 1: Machine learning variables

Variable name	Variable category	Number of variables extracted		Code	Data type	No. of variables with bivariate MOD association		Number of variables in final model	
		Dizz.	Abd.			Dizz.	Abd.	Dizz.	Abd.
Prior hospitalizations	Cohort	1	1		count				
Prior ED visits	Cohort	1	1		count				
ED duration	Cohort	1	1		numeric	1	1	1	1
Time from ED to inpatient admission	Cohort	1	1		numeric	1	1	1	1
Hospital length of stay	Cohort	1	1		numeric				
Systolic	Vital signs	8 *	8		numeric	3	2	2	2
Diastolic	Vital signs	8	8		numeric	3	3	3	3
Heart rate	Vital signs	8	8		numeric	4	4	4	4
Respiratory rate	Vital signs	8	8		numeric	2	1	2	1
Pain score	Vital signs	8	8		numeric		5		5
Temperature	Vital signs	8	8		numeric		6		6
Age at ED visit	Demographic	1	1	L288	numeric				
Gender	Demographic	1	1		boolean				
Ethnicity	Demographic	1	1		boolean		1		1
Race	Demographic	5 **	5		categorical				
Neurology consults	Consults in ED	1	0		count				
Cardiology consults	Consults in ED	1	0		count				
Gastroenterology consults	Consults in ED	0	1		count				
Endoscopy consults	Consults in ED	0	1		count				
General surgery consults	Consults in ED	0	1		count				
Chart consults (remote consultant review)	Consults in ED	0	1		count				

© 2024 Zimolzak AJ et al. *JAMA Network Open.*

Variable name	Variable category	Number of variables extracted		Code	Data type	No. of variables with bivariate MOD association		Number of variables in final model	
		Dizz.	Abd.			Dizz.	Abd.	Dizz.	Abd.
EKGs ordered	Misc	1	1		count				
CT scans	Radiology in ED	1	1	L632	count	1	1		1
CT scans with contract	Radiology in ED	1	1		count				
CT scans, abnormal	Radiology in ED	1	1		count	1	1		1
X-rays	Radiology in ED	1	1		count	1			
X-rays, abnormal	Radiology in ED	1	1		count				
MRIs	Radiology in ED	1	1		count				
MRIs, abnormal	Radiology in ED	1	1		count				
Ultrasounds	Radiology in ED	0	1		count				
Ultrasounds, abnormal	Radiology in ED	0	1		count				
White blood cells	Labs in ED	4 †	4	L808	numeric	1	1		1
Glucose	Labs in ED	4	4		numeric	4		4	
Albumin	Labs in ED	4	4		numeric	1			
Potassium	Labs in ED	4	4		numeric		1		1
Calcium	Labs in ED	4	4		numeric				
Lactate	Labs in ED	4	4		numeric				
Chloride	Labs in ED	4	4		numeric		1		1
Blood urea nitrogen	Labs in ED	4	4		numeric				
Creatinine	Labs in ED	4	4		numeric				
Troponin	Labs in ED	4	4		numeric				
Bicarbonate	Labs in ED	4	4		numeric	1			
AST	Labs in ED	4	4		numeric				
ALT	Labs in ED	4	4		numeric				
Alkaline phosphatase	Labs in ED	4	4		numeric	1			
Lipase	Labs in ED	4	4		numeric				

© 2024 Zimolzak AJ et al. *JAMA Network Open.*

		Number of variables extracted				No. of variables with bivariate MOD association		Number of variables in final model	
**Variable name**	**Variable category**	**Dizz.**	**Abd.**	**Code**	**Data type**	**Dizz.**	**Abd.**	**Dizz.**	**Abd.**
Amylase	Labs in ED	4	4		numeric		1		1
Hemoglobin	Labs in ED	4	4		numeric	1			
Cirrhosis	Past visit diagnoses	0	1	L888	count	na		na	
Cholecystitis	Past visit diagnoses	0	1		count	na	1	na	1
Cholelithiasis	Past visit diagnoses	0	1		count	na		na	
Appendicitis	Past visit diagnoses	0	1		count	na		na	
Diverticulitis	Past visit diagnoses	0	1		count	na		na	
Diverticulosis	Past visit diagnoses	0	1		count	na		na	
Inflammatory bowel disease	Past visit diagnoses	0	1		count	na		na	
Pancreatitis	Past visit diagnoses	0	1		count	na		na	
Stroke	Past visit diagnoses	1	0		count		na		na
Hypertension	Past visit diagnoses	1	0		count		na		na
Hyperlipidemia	Past visit diagnoses	1	0		count		na		na
Diabetes	Past visit diagnoses	1	0		count		na		na
Smoking	Past visit diagnoses	1	0		count		na		na
Atrial fibrillation	Past visit diagnoses	1	0		count		na		na
Occlusion/stenosis of cerebral artery	Past visit diagnoses	1	0		count		na		na
Coronary artery disease	Past visit diagnoses	1	0		count		na		na
Cerebral aneurysm	Past visit diagnoses	1	0		count	1	na	1	na
**TOTALS**		***148***	***151***			***27***	***31***	***18***	***31***

Notes:

***** Each vital sign is summarized as minimum, maximum, count, and first value; for the ED visit and inpatient stay. This is a total of 4 * 2 = 8 summary statistics per vital sign.

****** Race was coded as a one-hot collection of 5 Boolean variables.

© 2024 Zimolzak AJ et al. *JAMA Network Open.*

† Each lab value is summarized as count of lab results, count of abnormal lab results, minimum, and maximum.

Abbreviations: Dizz. = dizziness e-trigger, Abd. = abdominal pain e-trigger, MOD = missed opportunity in diagnosis.

In the four rightmost columns, cells with zeros are left blank for ease of reading.

© 2024 Zimolzak AJ et al. *JAMA Network Open.*
