# Supplementary material for: Machine Learning to Enhance Electronic Detection of Diagnostic Errors
Source: JAMA Netw Open. 2024 Sep 9;7(9):e2431982. doi: 10.1001/jamanetworkopen.2024.31982 (PMC11385053; doi:10.1001/jamanetworkopen.2024.31982)
Supplement: Supplement 2. — Data Sharing Statement [file jamanetwopen-e2431982-s002.pdf]

Data Sharing Statement

Zimolzak. Machine Learning to Enhance Electronic Detection of Diagnostic Errors. *JAMA Netw Open*. Published September 09, 2024. doi:10.1001/jamanetworkopen.2024.31982

Data
Data available: No
